# Clinical Significance of Serum Galectin-9 and Soluble CD155 Levels in Patients with Systemic Sclerosis

**DOI:** 10.1155/2018/9473243

**Published:** 2018-11-27

**Authors:** Mami Chihara, Miki Kurita, Yuki Yoshihara, Akihiko Asahina, Koichi Yanaba

**Affiliations:** Department of Dermatology, The Jikei University School of Medicine, Tokyo, Japan

## Abstract

Signaling through coinhibitory receptors downregulates the immune response to prevent excessive immune activation and maintain optimal immunity and tolerance. The aim of this study was to examine the levels of the soluble forms of coinhibitory receptors and their ligands, namely, galectin-9 (the ligand of T-cell immunoglobulin and mucin domain 3) and CD155 (the ligand of T cell immunoglobulin and immunoreceptor tyrosine-based inhibitory motif domain), and their association with clinical features in patients with systemic sclerosis (SSc). The serum levels of galectin-9 and soluble sCD155 were examined by enzyme-linked immunosorbent assays in patients with SSc, and the results were evaluated with respect to clinical features. Patients with SSc exhibited raised serum levels of galectin-9, but not sCD155. Serum galectin-9 levels were raised not only in patients with diffuse cutaneous SSc but also in patients with limited cutaneous SSc. Furthermore, serum galectin-9 levels correlated positively with the erythrocyte sedimentation rate. In addition, increased serum galectin-9 levels tended to be associated with higher mortality and serious organ involvement. These results suggest that galectin-9, but not CD155, may be involved in the pathogenesis of SSc. In addition, the measurement of serum galectin-9 levels could be used to predict serious organ involvement and high mortality in patients with SSc.

## 1. Background

Systemic sclerosis (SSc) is a generalized connective tissue disorder of unknown etiology and is characterized by excessive fibrosis and microvascular damage in the skin and various internal organs. A growing body of evidence has demonstrated that SSc is an autoimmune disorder because of the presence of disease-associated autoantibodies and a number of immunologic abnormalities [1]. In particular, cutaneous mononuclear cells that infiltrate early SSc skin lesions consist of mostly activated T cells [2]; the degree of cell infiltration correlates with the severity of skin thickening [3]. These infiltrating cells are suggested to release various cytokines, chemokines, or growth factors that play an important role in the initiation and development of fibrosis in SSc [4].

T cells are initially stimulated by signaling through antigen-specific T cell receptors, while optimal T cell activation further requires both stimulatory and inhibitory secondary signals through costimulatory and coinhibitory receptors, respectively [5, 6]. In particular, signaling through coinhibitory receptors downregulates the immune response to prevent excessive immune activation and maintain optimal immunity and tolerance [7]; therefore, coinhibitory receptors are considered to play a crucial role in autoimmunity [8]. Remarkably, the administration of the fusion protein of cytotoxic T lymphocyte-associated antigen 4, which is the most characterized coinhibitory receptor, improves SSc skin fibrosis in mice and humans [9, 10]. Furthermore, multiple soluble forms of coinhibitory receptors, including cytotoxic T lymphocyte-associated antigen 4, programmed death-1, and T-cell immunoglobulin and mucin domain 3 (TIM-3), have been demonstrated to play a role in the pathogenesis of SSc [11–13]. Therefore, we hypothesized that other soluble forms of coinhibitory receptors and their ligands, such as galectin-9 (the ligand of TIM-3) and CD155 (the ligand of T cell immunoglobulin and immunoreceptor tyrosine-based inhibitory motif domain), may also play a role in the pathogenesis of SSc. In this study, we examined the levels of serum galectin-9 and soluble sCD155 in patients with SSc and evaluated the results with respect to clinical features.

## 2. Material and Methods

### 2.1. Patients and Controls

For the measurement of serum galectin-9 and sCD155 levels, samples were obtained from 62 Japanese patients with SSc (60 women and 2 men; median age 49 (range, 9–78) years). All patients fulfilled the criteria for SSc [14, 15]. The patients were classified according to the system proposed by LeRoy et al. [16]: 33 patients had limited cutaneous SSc (lcSSc) and 29 had diffuse cutaneous SSc (dcSSc). Antitopoisomerase I (topo I) antibodies (Abs) were present in 23 patients, anticentromere Abs were present in 28, anti-U1RNP Abs were present in 2, and anti-RNA polymerase I and III Abs were present in 2. No patients were positive for anti-U3RNP Abs. The remaining 7 patients were negative for all of these Abs. Mean disease duration was 4.6 ± 5.0 (range, 0.2–20) years. Duration was calculated from the time of the onset of the first clear clinical manifestation of SSc (excluding Raynaud's phenomenon). None of the patients had received corticosteroids or other immunosuppressants. Twenty-six age- and sex-matched healthy Japanese individuals were included in the study as healthy controls.

### 2.2. Clinical Assessment

During their first visit, all patients underwent a physical examination, complete medical histories were obtained, and laboratory tests were performed. Organ system involvement was defined as described previously [4, 17–19]; lung: bibasilar fibrosis on chest radiography and high-resolution computed tomography; esophagus: hypomotility shown by barium radiography; heart: pericarditis, congestive heart failure, or arrhythmia requiring treatment; kidney: malignant hypertension and rapidly progressive renal failure with no other explanation; joints: inflammatory polyarthralgia or arthritis; and muscle: proximal muscle weakness and elevated serum creatine kinase level. Interstitial lung disease was defined as bibasilar interstitial fibrosis on chest high-resolution computed tomography. In addition, a pulmonary function test, including vital capacity (VC) and diffusion capacity for carbon monoxide (DLco), was performed to examine the severity of interstitial lung disease. DLco < 75% and VC < 80% of predicted normal values were considered abnormal. The presence of the elevated right ventricular systolic pressure of ≥40 mm Hg by Doppler echocardiogram was used as a screening threshold for pulmonary arterial hypertension. Pulmonary arterial hypertension was confirmed by right-sided heart catheterization and a mean pulmonary artery pressure of ≥25 mm Hg in conjunction with a pulmonary capillary wedge pressure of ≤15 mm Hg. Erythrocyte sedimentation rate (ESR) and C-reactive protein (CRP) were considered elevated when each value was higher than 15 mm/h and 0.3 mg/dl, respectively.

Patients with SSc who were smokers or had respiratory disorders that might have affected DLco or VC were excluded from this study. The modified Rodnan skin score was calculated as the sum of skin thickness measurements determined by palpation on a scale of 0 to 3 in 17 body areas [20]. The study protocol was approved by The Jikei University School of Medicine, Tokyo, Japan, and informed consent was obtained from all subjects.

### 2.3. Enzyme-Linked Immunosorbent Assay

Fresh venous blood samples were drawn into pyrogen-free blood collection tubes without additives, immediately immersed in melting ice, and allowed to clot for 1 h before centrifugation. All serum samples were stored at −70°C until use. The serum levels of galectin-9 and sCD155 were measured with a specific enzyme-linked immunosorbent assay kit from R&D Systems (Minneapolis, MN) and LifeSpan BioSciences (Seattle, WA), respectively, according to the manufacturers' protocols. Each sample was tested in duplicate. The detection limits of each assay were as follows: 3 pg/ml for galectin-9 and 2.20 pg/ml for sCD155.

### 2.4. Statistical Analysis

Data are presented as the mean ± standard deviation (SD). The Kruskal–Wallis test was used to compare the serum levels of galectin-9 and sCD155 between the groups, Fisher's exact probability test was used to compare frequencies, and Bonferroni's test was used for multiple comparisons. Spearman's rank correlation coefficient was used to examine the relationship between two continuous variables. Multiple linear regression analysis was used to assess the association between serum galectin-9 levels adjusted for age at onset. A probability (*P*) value < 0.05 was considered significant.

## 3. Results

### 3.1. Serum Levels of Galectin-9 and sCD155 in Patients with SSc

**Figure 1:**
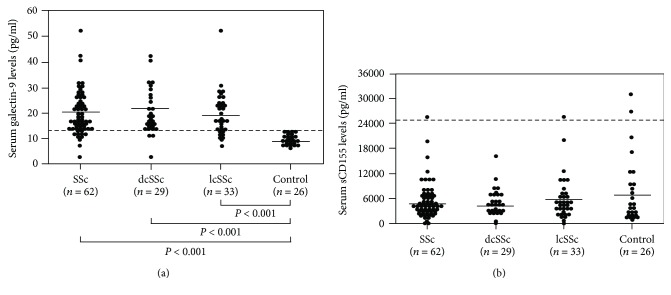
Comparison of groups according to (a) serum galectin-9 and (b) sCD155 levels. The control group comprised healthy individuals. The bars indicate the mean values in each group. The dashed line indicates the cut-off value (mean + 2 SDs of control values). SSc: systemic sclerosis; dcSSc: diffuse cutaneous SSc; lcSSc: limited cutaneous SSc.

The serum levels of galectin-9 and sCD155 in patients with SSc and healthy controls are shown in Figure 1. The mean serum galectin-9 levels at first visit were significantly higher in patients with SSc than in healthy individuals (20.4 ± 8.7 vs. 10.0 ± 1.8 pg/ml, respectively; *P* < 0.001). Among the SSc subgroups, galectin-9 levels were significantly increased in patients with dcSSc (21.0 ± 8.9 pg/ml) and those with lcSSc (19.8 ± 8.6 pg/ml) compared with healthy individuals (*P* < 0.001 for both). There was no significant difference in serum galectin-9 levels between patients with dcSSc and patients with lcSSc.

The mean serum levels of sCD155 at first visit were comparable between patients with SSc (5618 ± 4662 pg/ml) and healthy individuals (8592 ± 7835 pg/ml, respectively). With respect to the SSc subgroups, there was no significant difference in the serum levels of sCD155 between patients with dcSSc and patients with lcSSc. Thus, the serum levels of galectin-9, but not sCD155, were increased in patients with SSc.

### 3.2. Correlation between Clinical Features and Serum Galectin-9 Levels in Patients with SSc (Tables 1 and 2)

**Figure 2:**
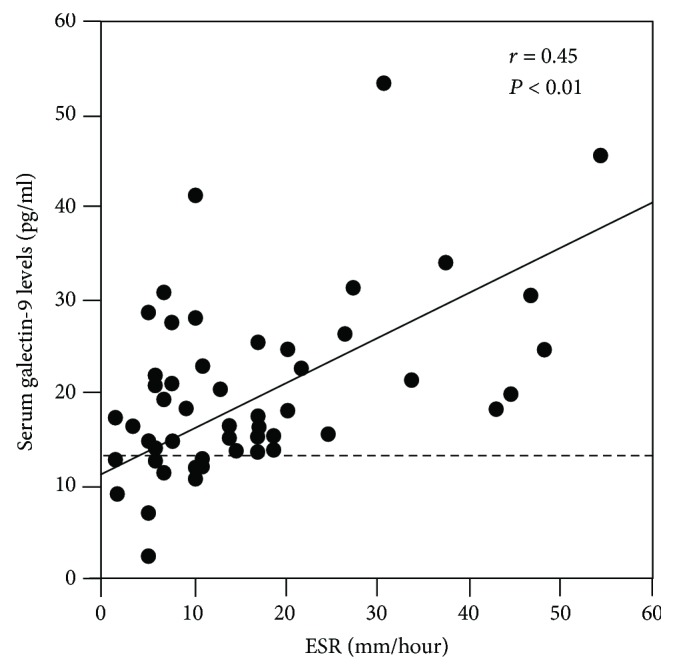
Correlation between serum galectin-9 levels and the ESR in patients with SSc. The dashed line indicates the cut-off value. ESR: erythrocyte sedimentation rate.

Clinical and laboratory parameters obtained at the first evaluation were compared between patients with SSc and increased galectin-9 levels and patients with SSc and normal galectin-9 levels. Values greater than the mean + 2 SDs (13.5 pg/ml) of control serum samples were considered to be increased in this study. Increased galectin-9 levels were observed in 82% (51/62) of all patients with SSc, 53% (27/51) of patients with dcSSc, and 47% (24/51) of patients with lcSSc. Furthermore, patients with SSc and increased galectin-9 levels had an elevated ESR more frequently than those with normal galectin-9 levels (41% vs. 0%, respectively; *P* = 0.01). Consistently, galectin-9 levels correlated positively with the ESR (*P* < 0.01, *r* = 0.45; Figure 2). Furthermore, this finding remained significant in multivariable analysis after adjustment for age at onset, suggesting that increased serum galectin-9 levels may have a greater impact on elevated ESR rather than age at onset.

The patients with SSc who died within 10 years after disease onset had higher serum galectin-9 levels (*n* = 4, 26.8 ± 7.0 pg/ml) than those who survived at least 10 years (*n* = 58, 20.0 ± 8.8 pg/ml), although the difference was not statistically significant (*P* = 0.05). Furthermore, all 4 SSc patients who died within 10 years after disease onset had increased serum galectin-9 levels (33.1, 31.1, 24.0, and 19.0 pg/ml). Therefore, increased serum galectin-9 levels may predict mortality in patients with SSc.

## 4. Discussion

In this study, patients with SSc exhibited raised serum galectin-9 levels, but not sCD155 levels. Serum galectin-9 levels were raised not only in patients with dcSSc but also in patients with lcSSc. Elevated galectin-9 levels correlated positively with the ESR. Furthermore, increased serum galectin-9 levels tended to be associated with higher mortality and serious organ involvement. These results suggest that galectin-9 may play an important role in the pathogenesis of SSc.

Galectin-9 is a ligand of TIM-3, is expressed highly on CD4^+^ T helper (Th) 1 and Th17 cells, but not on Th2 cells, and provides inhibitory signals through its interaction with TIM-3 [7]. Galectin-9 is upregulated in dermal fibroblast in patients with SSc [21]. In a bleomycin-induced mouse model of SSc, galectin-9 suppression using *Lgals9* small interfering RNA attenuates skin fibrosis, while it augments the expression of interferon-*γ*, leading to Th1-skewed immune polarization [21]. Considering that SSc is regarded as a Th2/Th17-polarized autoimmune disease [22, 23], increased circulating levels of galectin-9 may cause a Th2-predominant immune imbalance, thereby inducing the pathological progression of SSc.

Several reports have revealed that an elevated ESR is associated with a higher mortality rate in patients with SSc [24–27]. An elevated ESR has also been reported to be a predictor of pulmonary arterial hypertension [28]. Consistent with this, SSc patients with elevated serum galectin-9 levels tended to have the diffuse cutaneous subtype and vital organ involvement more frequently than those with normal serum galectin-9 levels, although the differences were not statistically significant. Thus, elevated serum galectin-9 levels may also be a predictor of serious organ involvement and high mortality in patients with SSc, similar to an elevated ESR.

There are several potential limitations of this study that should be considered. First, the patient population was relatively small; a larger study is essential to confirm our results. Second, all patients with SSc and healthy individuals in this study were Japanese; additional studies are needed to verify these findings in other ethnic groups. Third, the precise mechanism by which galectin-9 contributes to the pathogenesis of SSc was not clarified. Fourth, it will be important to examine the longitudinal changes of serum galectin-9 levels in patients with SSc and to assess their association with ESR, mortality, serious organ involvement, in particular, interstitial lung disease and pulmonary arterial hypertension, and disease activity in the future studies. Nevertheless, our findings suggest that galectin-9 may be involved in the pathogenesis of SSc. Moreover, we did not find a significant increase in serum sCD155 levels in patients with SSc in the current study; however, this does not necessarily exclude a role for these coinhibitory receptors in the pathogenesis of SSc.

## Figures and Tables

**Table 1:** Results of multiple linear regression analysis for ESR.

Predictive variable	Standard regression coefficient	*t* value	*P* value
Galectin-9 levels	0.428	3.390	0.001
Age at onset	0.208	1.652	0.105

ESR: erythrocyte sedimentation rate.

**Table 2:** Clinical and laboratory findings in patients with SSc according to serum galectin-9 levels.

	Elevated galectin-9	Normal galectin-9	*P* value
	*n* = 51	*n* = 11	
Age at onset, years (mean ± SD)	53 ± 15	46 ± 11	0.07
Male : female	2 : 49	0 : 11	>0.99
MRSS, points (mean ± SD)	10.2 ± 8.7	6.1 ± 5.6	0.27
Clinical features (%)			
dcSSc	53	27	0.19
lcSSc	47	73	0.19
Pitting scars/digital ulcers	37	27	0.73
Contracture of phalanges	47	36	0.74
Diffuse pigmentation	37	9	0.09
Telangiectasia	24	27	>0.99
Organ involvement (%)			
Interstitial lung disease	47	27	0.32
Decreased %VC	22	0	0.19
Decreased %DLco	47	27	0.32
Pulmonary hypertension	4	0	>0.99
Esophagus	63	91	0.43
Heart	10	0	0.58
Kidney	4	0	>0.99
Joint	25	18	>0.99
Muscle	20	9	0.67
Laboratory findings (%)			
Antitopoisomerase I Abs	39	27	0.52
Anticentromere Abs	43	55	0.52
Elevated ESR	41^∗^	0	0.01
Elevated CRP	12	0	0.58

Values were taken on the first visit. ^∗^*P* < 0.05 versus SSc patients with normal serum galectin-9 levels. SSc: systemic sclerosis; SD: standard deviation; MRSS: modified Rodnan skin score; dcSSc: diffuse cutaneous systemic sclerosis; lcSSc: limited cutaneous SSc; VC: vital capacity; DLco: diffusion capacity for carbon monoxide; Abs: antibodies; ESR: erythrocyte sedimentation rate; CRP: C-reactive protein.

## Data Availability

The data used to support the findings of this study are available from the corresponding author upon request.
